# High-resolution laser spectroscopy of singly charged natural uranium isotopes

**DOI:** 10.1038/s41598-024-76975-w

**Published:** 2024-10-27

**Authors:** Andrea Raggio, Michael Block, Paul Campbell, Bradley Cheal, Ruben P. de Groote, Wouter Gins, Ágota Koszorús, Iain D. Moore, Alejandro Ortiz-Cortes, Ilkka Pohjalainen, Jessica Warbinek

**Affiliations:** 1 Accelerator Laboratory, Department of Physics, University of Jyväskylä, Jyväskylä, 40014 Finland; 2 GSI Helmoltzzentrum für Schwerionenforschung GmbH, Planckstr.1, 64291 Darmstadt, Germany; 3 Department of Chemistry-TRIGA Site, University of Mainz, 55099 Mainz, Germany; 4 Helmholtz Institute Mainz, 55099 Mainz, Germany; 5 Department of Physics and Astronomy, University of Manchester, Manchester, M13 9PL UK; 6 Department of Physics, University of Liverpool, Liverpool, L69 7ZE UK; 7 Grand Accelerateur National d’Ions Lourds (GANIL), CEA/DSM-CNRS/IN2P3, Caen, France; 8 Present Address: KU Leuven, Instituut voor Kern- en Stralingsfysica, 3001 Leuven, Belgium; 9 Present Address: Facility for Rare Isotope Beams, Michigan State University, 630 South Shaw Lane East, Lansing, MI 48824 USA; 10 Present Address: Experimental Physics Department, CERN, 1211 Geneva 23, Switzerland

**Keywords:** Collinear laser spectrocopy, Actinides, IGISOL, Electronic structure of atoms and molecules, Experimental nuclear physics

## Abstract

High-resolution collinear laser spectroscopy has been performed on singly charged ions of $^{234,235,238}$U at the IGISOL facility of the Accelerator Laboratory, University of Jyväskylä, in Finland. Ten ionic transitions from the $^{4}\hbox {I}_{9/2}$ and $^{6}\hbox {L}_{11/2}$ ground and first excited states were measured in the 300 nm wavelength range, improving the precision of the hyperfine parameters of the lower states in addition to providing newly measured values for the upper levels. Isotope shifts of the analyzed transitions are also reported for $^{234,235}$U with respect to $^{238}$U.

## Introduction

The actinides include the 14 chemical elements of the 5f series with atomic numbers from *Z* = 89 to 102, and lawrencium with *Z* = 103. Unlike the lighter elements, all isotopes associated with the actinides are radioactive, with primordial isotopes of only thorium and uranium occurring naturally in substantial quantities. The actinides have always been of great research interest in part due to their numerous applications, but also because of their rich chemistry which gives rise to rather unique properties^1^ . The fingerprint of the atom, imprinted in the electronic structure, offers access to atomic level energies, transition strengths, lifetimes and the ionization potential. Traditionally, the main interest in atomic spectroscopy of the actinide elements has been in energy level analysis, with atomic levels and transitions representing a starting point for exploring the complex atomic structure^2^. Generally, the atomic structure of the majority of these elements is only partially known due to the scarcity of material, lack of stable isotopes and the difficulty in synthesizing macroscopic quantities, requiring nuclear reactions, rapid separation and identification^3^. These limitations are reflected in the dearth of ground state nuclear structure information from optical measurements which probe the shifts and splittings of the electronic energy levels, providing information on the nuclear charge distribution, electromagnetic moments and nuclear spin^4^. Fortunately, tremendous progress in the past decade has been made to develop highly sensitive and efficient techniques of laser spectroscopy^3^, highlighted in the pioneering studies of isotopes of nobelium (*Z* = 102) produced in atom-at-a-time quantities, the heaviest species to be studied with laser spectroscopy^5,6^.

Uranium is one of the most thoroughly studied actinide elements, in part due to its prominence in the field of nuclear energy and related applications. A total of 28 isotopes of uranium are known, primarily through nuclear decay spectroscopy^7^, with $^{241}$U the most recent isotope discovered, produced using multi-nucleon transfer reactions at the High Energy Accelerator Research Organization (KEK) in Japan^8^. Of these isotopes, only five have been explored with laser spectroscopy, namely $^{233-236,238}$U^9^, motivated by the study of nuclear shape evolution extracted through changes in mean-square charge radii. In spite of this study, there remains a lack of experimental data in the available literature on the hyperfine structure of both neutral and ionic uranium. Precise atomic calculations of energy levels and hyperfine constants are also extremely challenging due to the high number of valence electrons (5 and 6 for the singly charged and neutral state, respectively), high density of electronic states and the presence of relativistic effects^10,11^. In this context, precise measurements of spectroscopic quantities such as isotope shifts and hyperfine structure parameters are needed to refine and improve the accuracy of atomic calculations^10^. Moreover, $^{238}$U$^{+}$ has been identified as a possible cosmochronometer by measuring its abundance via high-resolution spectroscopy of metal poor stars^12^, requiring therefore a detailed knowledge of the ionic spectroscopy properties.

The use of laser spectroscopy techniques provides a gateway to unveil the distribution of electric charge and current in the nucleus^3,4,13–15^, and has been applied on both neutral^9,16–18^ and singly ionized uranium^19^. In this work, collinear laser spectroscopy has been employed in the measurement of ten transitions in the singly charged natural uranium isotopes, providing hyperfine parameters for $^{235}$U and isotope shifts for $^{234,235}$U with respect to $^{238}$U. These three isotopes are present in appreciable quantities in nature and are thus ideally suited for basic atomic structure studies. Our measurement data will also serve as a future reference for the study of the second lowest known isomeric state in the nuclear landscape, the 76-eV isomer $^{235m}$U^20^. This state exists due to a very fine interplay between collective-shape and intrinsic single-particle degrees of freedom. With a nuclear spin and parity of $1/2^{+}$, the isomer de-excites by a highly converted E3 transition to the ground state of $^{235}$U. Theoretical efforts to correctly predict the magnetic dipole moment of the lowest-lying isomer $^{229m}$Th^21^ employed a model that emphasized the role of collective quadrupole-octupole vibration-rotation motion, typical for actinide nuclei, coupled with the motion of an unpaired nucleon in a reflection-asymmetric deformed potential^22^. The authors of that work highlight $^{235m}$U as another interesting case for which an experimental measurement of the magnetic moment could further explore the applicability of the model.

## Experimental methodology

**Fig. 1:**
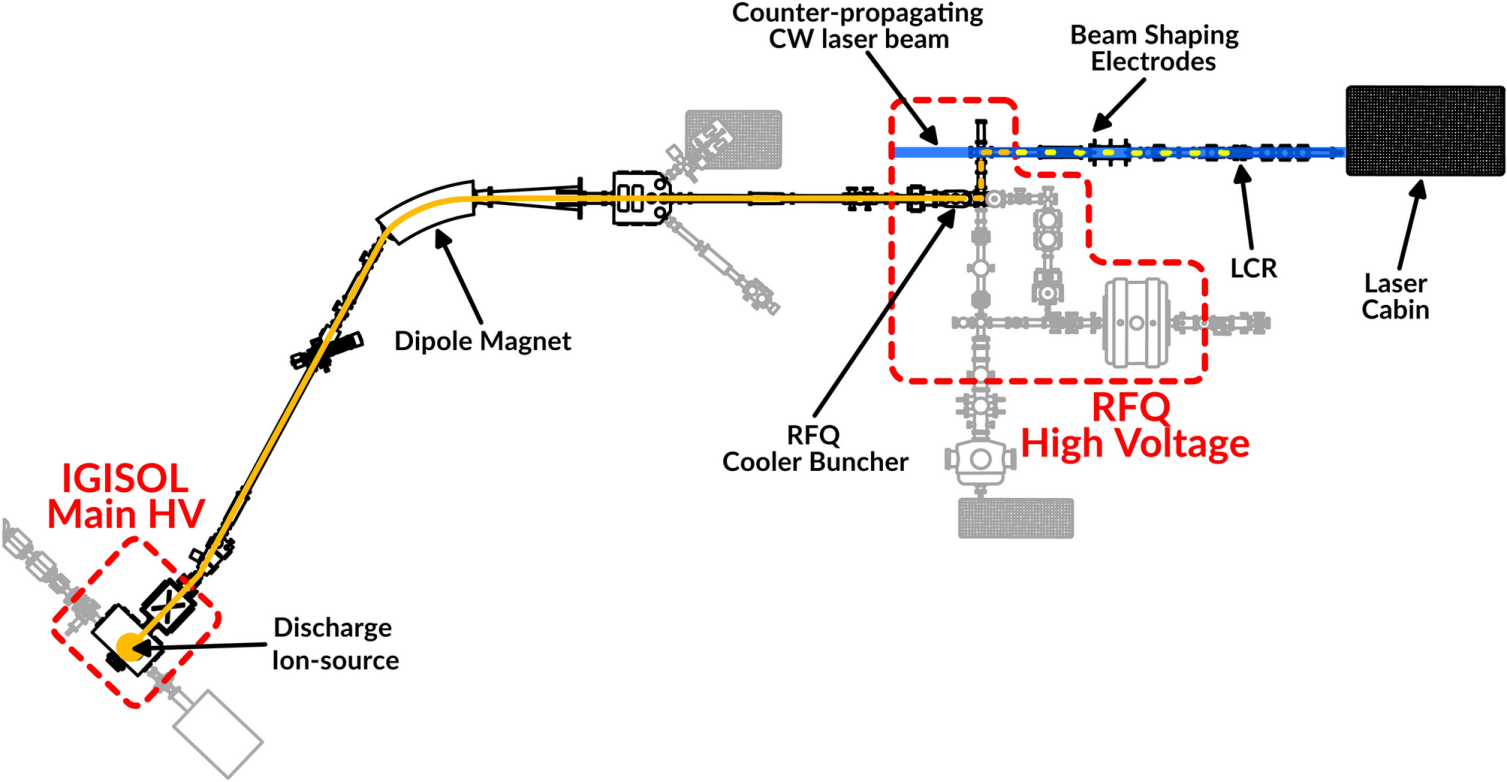
Schematic aerial view of the IGISOL facility. The two high voltage sections of the beamline are contoured in red dashed lines, while the $\sim 30$ keV beam produced by the discharge ion-source is represented by the orange continuous line. The bunched beam extracted from the RFQ is represented by orange dashed line in the 800 eV transfer section, and yellow dashed line when accelerated to the final $\sim 30$ keV energy before being injected into the collinear line. The CW counter-propagating laser beam is represented in blue. Other beamline sections and experiments present at IGISOL not relevant to this work are shaded in gray.

The work reported in this article was performed at the IGISOL (Ion Guide Isotope Separation On-Line) facility^23^, located at the Accelerator Laboratory of the University of Jyväskylä in Finland. A scheme of the experimental setup described in the following is presented in Fig. 1. An offline glow-discharge ion source equipped with a natural uranium electrode ($^{238}$U 99.284%, $^{235}$U 0.711%, $^{234}$U 0.0055%) was installed in the IGISOL target chamber. In this source, ions created during the discharge process are thermalized in a small helium volume at a pressure of $\sim$25 mbar. The ions are then extracted via gas flow and guided through a radiofrequency sextupole ion guide^24^ towards the high vacuum region of the mass separator. At this stage the ions are accelerated to a potential of 30 kV and are mass separated using a dipole sector magnet with a mass resolving power M/$\Delta$M$\approx 300$. The ions are then injected into a radiofrequency quadrupole (RFQ) cooler-buncher^25^. The RFQ is floated to a potential of approximately 100 V below the IGISOL high voltage (30 kV) and is filled with helium buffer gas at a pressure of $\sim 10^{-2}$ mbar to thermalize the incoming ions. Suitably applied potentials to the electrodes of the RFQ enable a controlled periodic release of cooled ions in a bunch with a few $\upmu$s time width and $\sim$1 eV energy spread. The cooling and release cycle can vary from a few ms to hundreds of ms depending on the requirements of the experiment and the incoming beam intensity. The ions are then extracted from the RFQ, accelerated to 800 eV and guided through two quadrupole benders, followed by a reacceleration to a final energy of 30 keV at the entrance to the collinear laser spectroscopy beam line.

A set of electrostatic deflector electrodes, extraction lenses and quadrupole electrodes are used to shape the beam and focus it to the laser–ion interaction region. In the conventional method of collinear laser spectroscopy applied here, the ion beam is overlapped with a counter-propagating continuous wave (cw) laser beam used to excite the ions on a chosen transition. A dedicated chamber surrounding the interaction region hosts a Hamamatsu 5900 L16 segmented Photomultiplier Tube (PMT) used to detect the fluorescence de-excitation photons emitted by the isotope of interest. This part of the setup is referred to as the Light Collection Region (LCR). The optimal alignment between the ion- and laser beam is ensured by the use of a movable 1 mm aperture hole located at the LCR. An ETP magneTOF mini detector for single ion counting and a Faraday cup are available downstream and are used alternately for beam intensity monitoring and tuning. The laser light is generated by one of two Sirah Matisse-2 lasers pumped by a 20 W Spectra Physics Millenia eV laser. The output wavelength is locked to the readout of a HighFinesse WSU-10 wavelength meter. The use of 30 keV beams results in a compression of the velocity spread of the ionic ensemble, producing a reduction of the Doppler broadening in the resulting spectral linewidths. The application of so-called bunched beam collinear laser spectroscopy allows for a time gating of the PMT signals at the time of flight of the ions of interest from the RFQ to the interaction region, producing a reduction of laser scatter background of up to 4 orders of magnitude while preserving the fluorescence signal^25,26^.

In the typical operation mode the laser is kept at a fixed wavelength while the velocity of the ion beam is tuned by applying a voltage to the LCR. This is achieved by the use of the 16-bit analog output of a Measurement Computing USB-3102 data acquisition board which provides a low voltage signal in the range −10 V to 10 V. The signal is then amplified by a x1000 TREK 609E-6 high-voltage amplifier. In order to monitor the potential of the ions with high precision, two Keysight 34465 digital multimeters are implemented, one reading the LCR voltage through a 1:1000 voltage divider, while the other monitoring the RFQ cooler-buncher potential through an Ohm-Lab KV-30A 1:10000 voltage divider. Before each measurement a calibration is routinely performed on the LCR scanning voltage. More details about the data acquisition system and the collinear spectroscopy line in general can be found in Ref.^27^.

Within this work a set of 10 transitions in singly charged $^{234,235,238}$U$^{+}$ was investigated in a wavelength range suitable for both the laser system and the sensitivity of the PMT. The laser light was produced using a Sirah Matisse 2 DS, using either Rhodamine 610 or Rhodamine 590 dyes in order to produce the fundamental wavelength in the required ranges. This laser light was then frequency doubled using a Sirah Wavetrain 2 frequency doubling cavity. The laser frequency set point was chosen considering a 30 keV beam energy, to allow for the hyperfine structure of the isotopes to fit within the dynamic range of the scanning voltage (typically ± 5 kV). As a result, measurement of the $^{234,235,238}$U$^{+}$ isotopes can be performed without a laser frequency change thus reducing possible systematic errors (20 MHz due to the uncertainty of the WSU-10 wavelength meter^28^ in the second harmonic). A list of wavelengths, corresponding energy levels, laser set points and choice of dye is reported in Table 1. Each transition was investigated by first measuring the resonant fluorescence photons as a function of laser power using the abundant isotope $^{238}$U to obtain a measure of the power needed to saturate the transition. The saturation power, typically $\sim$1 mW, was then used for the study of $^{235}$U and $^{234}$U. The spectroscopic transition efficiencies were also computed using $^{238}$U from the ratio of detected photons per incoming beam intensity when the ions are on resonance with the ionic transition. This value ranges from 1 photon per 3000 ions (transition (i)) to 1 photon per 3$\cdot 10^{6}$ ions (transition (a)) for the transitions reported.

For each transition the hyperfine spectrum of $^{235}$U and the single resonance peak of $^{234,238}$U were measured. During the scan of the hyperfine structures, especially in the case of the transitions originating from the 289.041 $\hbox {cm}^{-1}$ metastable level where the structure width can be more than 10 GHz, multiple scanning ranges were set to scan only the frequencies of interest and to increase the measurement speed. For both $^{235}$U and $^{234}$U isotopes, the limited mass separation of the IGISOL dipole magnet at high *A* values and the higher natural abundance of $^{238}$U allowed the simultaneous measurement of $^{238}$U providing a concurrent reference for the isotope shift determination.

**Table 1:** Information on the measured $\hbox {U}^{+}$ transitions. The energy levels and atomic spins involved in the transitions are provided. The transitions intensities are reported from^29^.

ID	Transition wavelength [nm]	A-value [$\hbox {s}^{-1}$]	Lower level [$\hbox {cm}^{-1}$]	J	Upper level [$\hbox {cm}^{-1}$]	J	Fundamental set point [$\hbox {cm}^{-1}$]	Dye
**a**	288.910	2.369$\cdot {10}^{7}$	0.0	9/2	34,612.820	9/2	17,297.67	R. 590
**b**	289.047	6.252$\cdot {10}^{7}$	289.041	11/2	34,885.475	11/2	17,289.26	R. 590
**c**	290.366	4.376$\cdot {10}^{6}$	0.0	9/2	34,439.332	11/2	17,210.90	R. 590
**d**	291.343	8.736$\cdot {10}^{6}$	289.041	11/2	34,612.820	9/2	17,153.00	R. 590
**e**	291.511	1.548$\cdot {10}^{7}$	289.041	11/2	34,593.120	11/2	17,143.16	R. 590
**f**	286.652	5.003$\cdot {10}^{7}$	0.0	9/2	34,885.475	11/2	17,433.95	R. 590
**g**	286.131	3.068$\cdot {10}^{7}$	0.0	9/2	34,949.080	7/2	17,465.60	R. 590
**h**	303.896	7.140$\cdot {10}^{6}$	0.0	9/2	32,905.985	9/2	16,444.65	R. 610
**i**	305.108	2.110$\cdot {10}^{7}$	0.0	9/2	32,775.234	9/2	16,379.35	R. 610
**j**	312.224	5.651$\cdot {10}^{6}$	0.0	9/2	32,028.319	7/2	16,399.30	R. 610

## Data analysis

The fluorescence spectra obtained as a function of LCR voltage ($V_{\text {LCR}}$) were first converted to the ions’ rest frame laser frequency ($\nu$) using the following equation for an anti-collinear geometry between ion and laser beam^15^:

1 $$\begin{aligned} \nu = \nu _{\text {LSP}}\sqrt{\dfrac{1+\beta }{1-\beta }}, \quad \beta =\sqrt{1-\left( \dfrac{mc^2}{E_k + mc^2}\right) ^2}, \quad E_k = q\left( V_{\text {COOL}}-V_{\text {LCR}}\right) \end{aligned}$$

where $\nu _{\text {LSP}}$ is the laser frequency set point used during the measurement, *m* the ion mass, *q* the charge and $V_{\text {COOL}}$ the RFQ cooler-buncher voltage.

**Fig. 2:**
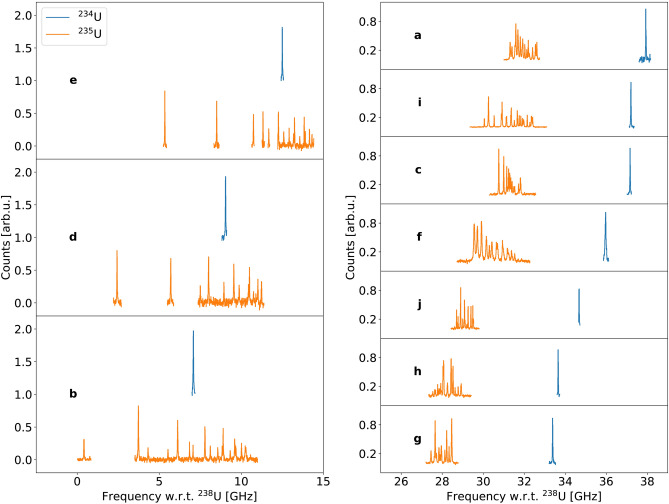
$^{234}$U and $^{235}$U measured spectra for each studied transition. The spectra are labeled according to the numbering of the transitions in Table 1. The three spectra on the left represent the transitions from the 289.041 $\hbox {cm}^{-1}$ ionic level, while on the right are the transitions originating from the ground state. An offset was added to the $^{234}$U spectra on the left with respect to the $^{235}$U ones for visualization purposes. All the resonances are plotted with respect to the corresponding $^{238}$U centroid (not shown in the picture for clarity).

For each transition, multiple scans were performed on each isotope, the measurements were then summed together by shifting each spectrum with respect to the $^{238}$U reference acquired either simultaneously or closest in time. The converted spectra are presented in Fig. 2 where the hyperfine structures and single resonance peaks of $^{235}$U and $^{234}$U with respect to the reference $^{238}$U are shown for each investigated transition.

The hyperfine structure of $^{235}$U originates from the nuclear spin *I* coupling to the atomic total angular momentum *J*. The energy shifts of the hyperfine levels from the fine structure energy are determined by^30^:

2 $$\begin{aligned} \Delta E = A\dfrac{K}{2} + B\dfrac{3K (K+1) - 4I(I+1)J(J+1)}{8I(2I-1)J(2J-1)}, \quad K=F(F+1)-J(J+1)-I(I+1) \end{aligned}$$

where *F* is the total angular momentum. In general, a hyperfine transition occurs between an *F* state of the upper fine structure level and an *F* state of the lower fine structure level, with the selection rules $\Delta F = 0,\pm 1$ and $0\not \rightarrow 0$. *A* and *B* are respectively the magnetic dipole and electric quadrupole hyperfine structure parameters, which are linked to the nuclear moments $\mu$ and $Q_s$ through the relations^4^:

3 $$\begin{aligned} A = \dfrac{\mu B(0)}{IJ}\quad \textrm{and} \quad B=eQ_s V_{zz}. \end{aligned}$$

Here, *e* is the electric charge, *B*(0) the magnetic field at the site of the nucleus and $V_{zz}$ the electric field gradient generated by the electrons at the nucleus.

To extract the *A* and *B* parameters for $^{235}$U and the isotope shifts for both $^{235}$U and $^{234}$U, the SATLAS fitting package was used^31^. The spectral lines were modeled using a Voigt profile implemented in the analysis library. In the case of $^{234}$U and $^{238}$U, the single resonance lines were fitted using a $\chi ^2$ minimization to extract their centroid values. An additional random walk sampling analysis based on the SATLAS implementation of the Markov Chain Monte Carlo (MCMC) algorithm was performed to have a better parameter error estimate and to monitor correlations between the hyperfine parameters. The hyperfine structure spectra of $^{235}$U were similarly fitted for each transition using a $\chi ^2$ minimization. The relative amplitudes of the different hyperfine components were left as free parameters in the fitting procedure. In the case of two or more peaks with a smaller separation than their full-width-at-half-maximum (FWHM) values, typically in the order of $\sim 20$ MHz, their amplitude ratio was constrained to Racah intensities, which generally are in good agreement with the experimental values. Since the hyperfine structures of transitions with the same upper or lower state share the same *A* and *B* parameter for that level, a fitting cost function was generated by combining together the ten transition fit models, initialized with their initial fit results. A global 248 parameter fit was then performed using shared *A* and *B* parameters over the whole dataset. Similar to the analysis of $^{234}$U and $^{238}$U, the random walk procedure was also applied.

**Fig. 3:**
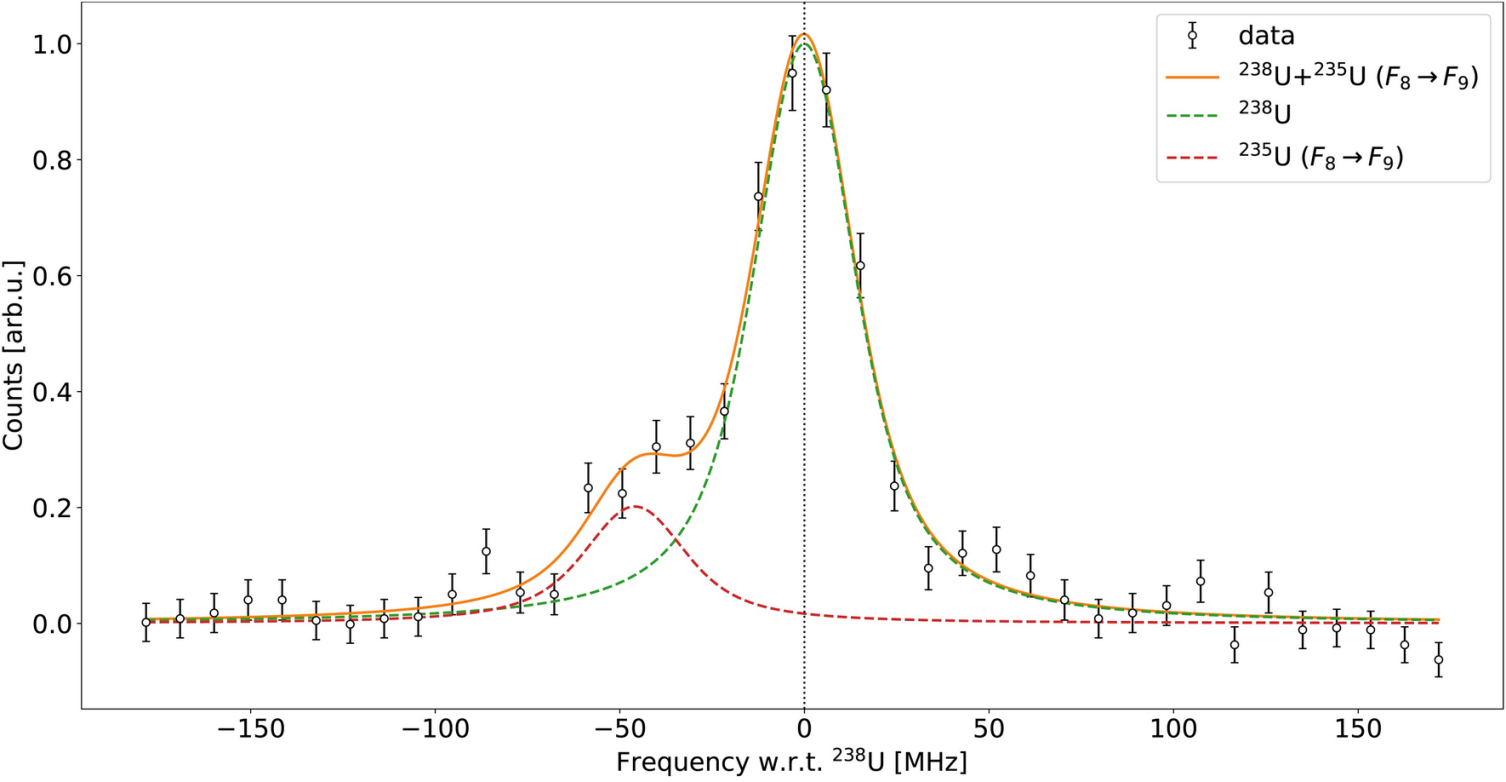
$^{238}$U spectrum of the 289.047 nm transition. The $F_8 \rightarrow F_9$ hyperfine component of $^{235}$U (dashed red line) partially overlapping with the $^{238}$U resonance (dashed green line) is presented in the figure, together with the total fitting function (solid orange line) used to extract the $^{238}$U reference centroid.

In the case of the 289.047 nm transition (panel b in Fig. 2), coincidentally the $F_8 \rightarrow F_9$ hyperfine line of $^{235}$U happens to be located $\sim$50 MHz from the $^{238}$U reference centroid due to the different kinematic shifts. Due to the limited mass resolving power of the mass separator dipole magnet, a fraction of $^{238}$U leaked through the separator with the dipole magnet set point *A*/*q* = 235 as previously described, and thus both peaks need to be accounted for in the fitting procedure. For this case, the data and fitting of $^{238}$U is presented in Fig. 3. During the $^{235}$U fitting procedure the aforementioned hyperfine component was not included in the fit, since the structure was already fully constrained by the other 21 hyperfine lines.

**Fig. 4:**
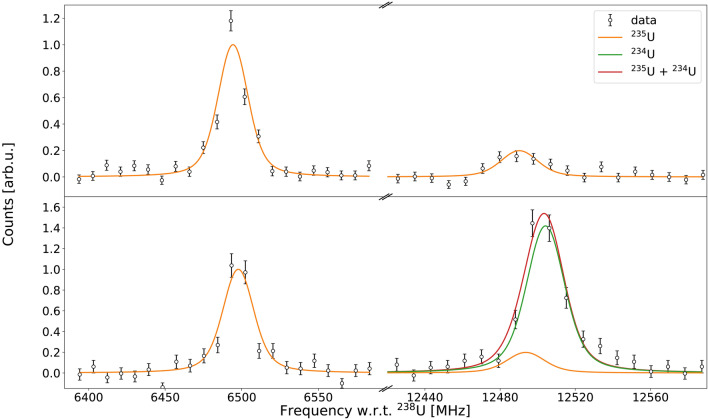
Spectra of the 291.511 nm transition in $^{235}$U, illustrating the $F_9 \rightarrow F_9$ (top left) and $F_7 \rightarrow F_6$ (top right) hyperfine lines obtained with the dipole magnet set point *A*/*q* = 235 and the same spectra obtained with the set point *A*/*q* = 234 (bottom). In the latter, the $^{234}$U resonance appears above the $F_7 \rightarrow F_6$ line (bottom right). The two spectra are converted to frequency using the $^{234}$U kinematic shift, and are normalized to the $F_9 \rightarrow F_9$ line intensity for comparison.

Similarly, in the 291.511 nm transition (panel e in Fig. 2), the $F_7 \rightarrow F_6$ hyperfine line of $^{235}$U falls within the FWHM of the $^{234}$U resonance when the dipole magnet set point is at *A*/*q* = 234. In order to compute an accurate $^{234}$U isotope shift, two different acquisitions were performed with the magnet set point at *A*/*q* = 235 and *A*/*q* = 234 with scanning ranges around the known positions of $F_7 \rightarrow F_6$ and $F_9 \rightarrow F_9$ $^{235}$U hyperfine components. When the magnet is driven towards the former set point the $^{234}$U contribution can be considered negligible due to the two orders of magnitude lower isotopic abundance with respect to $^{235}$U, allowing the relative position and intensities of the two hyperfine components to be extracted. Using this information and the leaking $F_9 \rightarrow F_9$ component with the latter magnet set point it is therefore possible to predict the $F_7 \rightarrow F_6$ transition that is present below the $^{234}$U resonance. The two acquisitions and the fits performed on the data are presented in Fig. 4.

## Results and discussion

The dipole and quadrupole hyperfine parameters extracted from the global fit are reported in Table 2 for each level. The literature values^19^ belonging to the ground state and the first excited state at 289.041 $\hbox {cm}^{-1}$ are reported next to those determined in this work. A good agreement is in general noticed, with the exception of the ground state dipole hyperfine parameter, for which a $\sim 3.6\sigma$ deviation is observed. We note that the independent fits of the seven transitions starting from this state produce values in agreement with each other in addition to the estimate obtained from the global fitting procedure, supporting the final (higher precision) value in our work.

For each hyperfine parameter the statistical uncertainty obtained from the random walk analysis is presented in parentheses. An additional systematic uncertainty is also shown in square brackets, originating from a 10 V systematic error present on the RFQ cooler-buncher voltage. A calibration measurement to assess the magnitude of this contribution was performed immediately prior to this experiment using stable Yb isotopes, whose hyperfine parameters are known with very high accuracy from microwave-optical double-resonance measurements^32^. The effect of this systematic error on the hyperfine spectra is discussed in more detail in Ref.^33^.

Considering the high precision of the obtained parameters, the fitting procedure described above was repeated with the introduction of the magnetic-octupole hyperfine interaction term *C* which appears as the next order contribution in Equation 2. No obvious changes to the *A* and *B* parameters were observed with the inclusion of this term. In order to assign a value to the magnetic-octupole term, second order hyperfine structure perturbations need to be quantified, however this is considered to be beyond the scope of this article.

To extract the hyperfine field coefficients reported in the final two columns of Table 2 from Equation 3, a magnetic dipole moment of −0.393(35) $\mu _N$ and electric quadrupole moment of 4.934(26) b were adopted, computed as a weighted average of the reported literature values^18,19,34–36^.

Lastly, the isotope shifts of $^{234}$U and $^{235}$U with respect to $^{238}$U are reported in Table 3. The isotope shift ratios shown for each transition in the last column of the table are found to be in agreement with the literature value of 0.8392(26)^37^. The small fluctuations among the calculated ratios, in the order of $10^{-4}$, are believed to be caused by the neglected mass shift contributions, which could not be quantified for the transitions studied in this work. Following this approximation in the case of very heavy nuclei with mass A>200, the isotope shift is directly proportional to the change in the mean-square charge radii, ${\delta \!\left\langle r_\textrm{c}^2 \right\rangle }$ = $\delta \nu /F$, where *F* is the electronic field factor of the corresponding transition. This atomic factor can be directly extracted from respective isotope shift measurements by applying a so-called King plot procedure^38^, which utilizes known model-dependent rms radii of uranium isotopes from muonic atom data measurements^39^. However, due to insufficient isotopes studied in this work, such a procedure cannot be employed. In the future, additional short-lived exotic isotopes of uranium may be available in sufficient quantities for collinear laser spectroscopy using online light-ion induced fusion-evaporation reactions. Such reactions have already been successfully demonstrated at IGISOL for the production and study of neutron-deficient isotopes of other actinide elements^40^. Additionally, theoretical developments for the determination of the electronic field factor would be welcome.

**Table 2:** Hyperfine structure parameters extracted from the fit of the studied transitions of $\hbox {U}^{+}$. Statistical uncertainties are reported in parentheses, while systematic uncertainties are given in square brackets. The last two columns reports the field constants computed using the literature dipole and quadrupole moments.

Level [$\hbox {cm}^{-1}$]	J	Term	*A* [MHz]		*B* [MHz]		A/$\mu$ [MHz/$\mu _N$]	B/Q [MHz/b]
			This work	Literature	This work	Literature		
0.000	9/2	$^{4}\hbox {I}_{9/2}$	$-$73.265(26)[11]	$-$76.9(10)^19^	1013.6(9)[3]	1017(50)^19^	186(17)	205.4(11)
289.041	11/2	$^{6}\hbox {L}_{11/2}$	146.17(4)[2]	146.3(7)^19^	4707.0(20)[5]	4740(30)^19^	$-$372(33)	954(5)
32028.319	7/2		$-$108.019(31)[12]		860.4(8)[1]		275(25)	174.4(9)
32775.234	9/2		$-$126.396(24)[11]		385.0(9)[2]		321(29)	78.0(4)
32905.985	9/2		$-$68.15(4)[1]		$-$33.8(16)[5]		173(16)	$-$6.86(34)
34439.332	11/2		$-$80.402(30)[11]		804.2(15)[2]		204(18)	163.0(9)
34593.120	11/2		$-$79.91(5)[1]		1501.4(29)[5]		203(18)	304.3(17)
34612.820	9/2		$-$91.646(27)[12]		1126.4(11)[4]		233(21)	228.3(12)
34885.475	11/2		$-$100.00(4)[2]		1567.9(21)[5]		254(23)	317.8(17)
34949.080	7/2		$-$102.00(4)[1]		$-$114.9(14)[3]		259(23)	$-$23.29(32)

**Table 3:** Isotope shifts for $^{234}$U and $^{235}$U measured for each transition with respect to $^{238}$U. All wavelengths are in vacuum. Statistical uncertainties are reported in parentheses, while systematic uncertainties are in square brackets.

ID	Transition wavelength [nm]	$\delta \nu ^{235-238}$ [MHz]	$\delta \nu ^{234-238}$ [MHz]	$\delta \nu ^{235-238}$/$\delta \nu ^{234-238}$	
**a**	288.910	31,850.0(5)[22]	37,929.7(7)[24]	0.839711(20)	
**b**	289.047	5936.6(9)[1]	7080.5(10)[3]	0.83845(17)	
**c**	290.366	31,203.8(4)[21]	37,163.3(4)[23]	0.839641(14)	
**d**	291.343	7582.7(12)[2]	9043.9(12)[1]	0.83844(18)	
**e**	291.511	10,495.4(10)[3]	12,503.6(12)[3]	0.83939(12)	
**f**	286.652	30,195.0(9)[21]	35,965.5(12)[23]	0.83955(4)	
**g**	286.131	28,033.6(7)[19]	33,384.4(6)[20]	0.839721(27)	
**h**	303.896	28,252.9(4)[19]	33,653.7(5)[21]	0.839520(17)	
**i**	305.108	31,249.8(3)[21]	37,202.3(4)[24]	0.839995(14)	
**j**	312.224	29,107.2(3)[19]	34,674.7(4)[22]	0.839435(14)	
				This work (Avg.)	Lit.
				0.83966(7)	0.8392(26)^37^

## Conclusion

High-resolution collinear laser spectroscopy has been performed on the naturally abundant $^{234,235,238}$U isotopes on 10 ionic transitions from the $^{4}\hbox {I}_{9/2}$ and $^{6}\hbox {L}_{11/2}$ ground and low-lying states in the 300 nm wavelength range. A global parameter fitting procedure was employed for the analysis of the spectra of $^{235}$U. From this work, 8 new magnetic dipole and electric quadrupole hyperfine parameters were extracted for the high lying levels in the 32,000–35,000 $\hbox {cm}^{-1}$ range, while the parameters for the ground state and first low lying state have been determined with an order of magnitude improvement in precision with respect to the literature values. Additionally, we present new isotope shift values for $^{234,235}$U with respect to $^{238}$U.

The new high-precision atomic physics data will contribute to further developments in atomic theory, benchmarking modern methods being applied to these challenging heavy elements. The newly extracted information will additionally serve as a reference for the future laser spectroscopy investigation of the $^{235m}$U 76-eV isomeric state, where the direct comparison of the *A* parameters and isomeric shifts will allow the extraction of the magnetic dipole moment and charge radius of the isomer. Efforts are currently underway to produce the isomeric state at IGISOL.

## Data Availability

The datasets generated and analysed during the current study are available in the Zenodo repository, https://doi.org/10.5281/zenodo.12671588.
